# Algae-Based Supplements Claiming Weight Loss Properties: Authenticity Control and Scientific-Based Evidence on Their Effectiveness

**DOI:** 10.3390/md22030123

**Published:** 2024-03-05

**Authors:** Fátima Fernandes, Raquel Martins, Mariana Barbosa, Patrícia Valentão

**Affiliations:** 1 REQUIMTE/LAQV, Laboratório de Farmacognosia, Departamento de Química, Faculdade de Farmácia, Universidade do Porto, Rua de Jorge Viterbo Ferreira n.º 228, 4050-313 Porto, Portugal; up201904818@ff.up.pt; 2 Associate Laboratory i4HB—Institute for Health and Bioeconomy, NOVA School of Science and Technology, Universidade NOVA de Lisboa, 2829-516 Caparica, Portugal; mn.barbosa@fct.unl.pt

**Keywords:** algae, *Fucus vesiculosus*, spirulina, food supplements, anti-obesity, antioxidant, quality control

## Abstract

The worldwide prevalence of obesity impacts more than 600 million adults. Successfully managing weight is effective in reducing the risk of chronic diseases, but sustaining long-term weight loss remains a challenge. Although there are supplements based on algae that claim to aid in weight loss, there is a notable scarcity of scientific evidence supporting their effectiveness, and their regular consumption safety remains inadequately addressed. In this work, commercially available *Arthrospira* (Spirulina) *platensis* Gomont and/or *Fucus vesiculosus* L. supplements showed moderate capacity to inhibit the activity of carbohydrate-metabolizing enzymes, and to scavenge biologically relevant reactive species. IC_25_ values varying between 4.54 ± 0.81 and 66.73 ± 5.91 µg of dry extract/mL and between 53.74 ± 8.42 and 1737.96 ± 98.26 µg of dry extract/mL were obtained for α-glucosidase and aldose reductase, respectively. A weaker effect towards α-amylase activity was observed, with a maximum activity of the extracts not going beyond 33%, at the highest concentrations tested. Spirulina extracts showed generally better effects than those from *F. vesiculosus*. Similar results were observed concerning the antiradical capacity. In a general way, the extracts were able to intercept the in vitro-generated reactive species nitric oxide (^•^NO) and superoxide anion (O_2_^•−^) radicals, with better results for O_2_^•−^scavenging with the spirulina samples (IC_25_ values of 67.16 and 122.84 µg of dry extract/mL). Chemically, similar pigment profiles were observed between spirulina supplements and the authenticated counterpart. However, fucoxanthin, the chemotaxonomic marker of brown seaweeds, was not found in *F. vesiculosus* samples, pointing to the occurrence of a degradation phenomenon before, during, or after raw material processing. Our findings can contribute to providing data to allow regulatory entities (e.g., EFSA and FDA) to better rule these products in a way that can benefit society.

## 1. Introduction

The global epidemic of obesity has impacted over 600 million adults [1]. Alongside the escalation in obesity rates, there has been a notable uptick in the prevalence of physiological comorbidities, encompassing hyperlipidemia, hyperglycemia, and chronic inflammation. These interconnected factors have played a role in the heightened occurrence of cardiovascular diseases and type 2 diabetes, both ranking among the foremost causes of mortality worldwide [1]. While successfully managing weight proves effective in reducing the risk of chronic diseases, the enduring maintenance of weight loss poses a significant challenge [2]. Recognizing the scale of this issue, it becomes imperative to pinpoint potential pathophysiological factors associated with obesity to formulate effective prevention and treatment strategies. Oxidative stress emerges as a common denominator in many obesity-related disorders and may serve as the underlying mechanism for the onset and progression of these conditions. Indeed, numerous studies have established a correlation between biomarkers of oxidative stress and obesity [3,4].

The marine environment represents one of the most dynamic arenas for research, capturing the scientific community’s attention due to its biodiversity, considered an infinite reservoir of unique and biologically active chemical structures [5]. The extensive variety of algae species, encompassing both macro and microalgae, yields a plethora of compounds with significant potential for human health. The growing utilization of algae in human food is becoming more apparent and is endorsed by both the European Food Safety Authority (EFSA) and the Food and Drug Administration (FDA) [6].

In addition to their direct consumption, many food supplements with algae species claim therapeutic purposes [7]. In fact, algae-based supplements have gained attention among the population, especially for weight loss, an effect strongly associated with a low-calorie content together with nutrient and protein richness. Potential acting mechanisms, including metabolism boost, blood sugar regulation, detoxification, and reduced fat absorption are endorsed to the supplement components [7]. Although there are several commercially available algae-based supplements indicating weight loss properties, scientific evidence on their effectiveness is still scarce and does not consider the safety of the regular and continuous consumption of these products. Food supplement legislation falls in a gray zone of foods, allowing its free consumption, but with bioactivities strong enough to be considered medicines, which should restrict their generalized consumption. The primary challenge in regulating dietary supplements is the lack of international consensus on how this category of products is defined, and standards that ensure quality and integrity do not exist in the global context [8].

In Portugal, several algae-based supplements are widely commercialized by several food supplement distributors, being commonly sold in herbal shops. Nevertheless, as far as we know, there are no studies about the authenticity of what is sold and about their effectiveness on weight control. We questioned whether these claims are scientifically supported. Focused on that, the main objectives of our work were (*i*) to assess parameters able to identify authenticity and efficacy of commercial algae-based supplements indicated for weight loss and (*ii*) to assess the effect of the procedure followed by the supplier, from production to selection, transformation, and conservation of algae and algae-based supplements, on their chemical composition and biological properties. The pigment profile, established by HPLC-DAD, was used as a “fingerprint” for authenticity control of the selected algae-based supplements. The supposed weight loss properties of the algae-supplements were assessed by evaluating their capacity to inhibit the activity of enzymes involved in the metabolism of carbohydrates (α-amylase and α-glucosidase) and of glucose (aldose reductase). The antiradical potential of the algae-based supplements, as well as of algae specimens was evaluated in vitro by the scavenging capacity against biologically relevant radicals, namely superoxide anion (O_2_^•−^) and nitric oxide (^•^NO) radicals.

## 2. Results and Discussion

### 2.1. Pigment Fingerprint

The lack of studies, legislation, and quality control of algae-based food supplements raise a series of problems, first and foremost on the authenticity of what is sold. Misidentification of the raw material for algae-based supplements can pose a serious concern. The presence of some secondary metabolism products, as well as of some carotenoids and chlorophylls in specific algal divisions or classes constitute important chemotaxonomic markers [9,10]. Thus, considering the taxonomic interest of these metabolites, pigment composition was used herein as a fingerprint of the marketed algae-based products.

#### 2.1.1. Spirulina and Spirulina-Based Supplements

**Figure 1:**
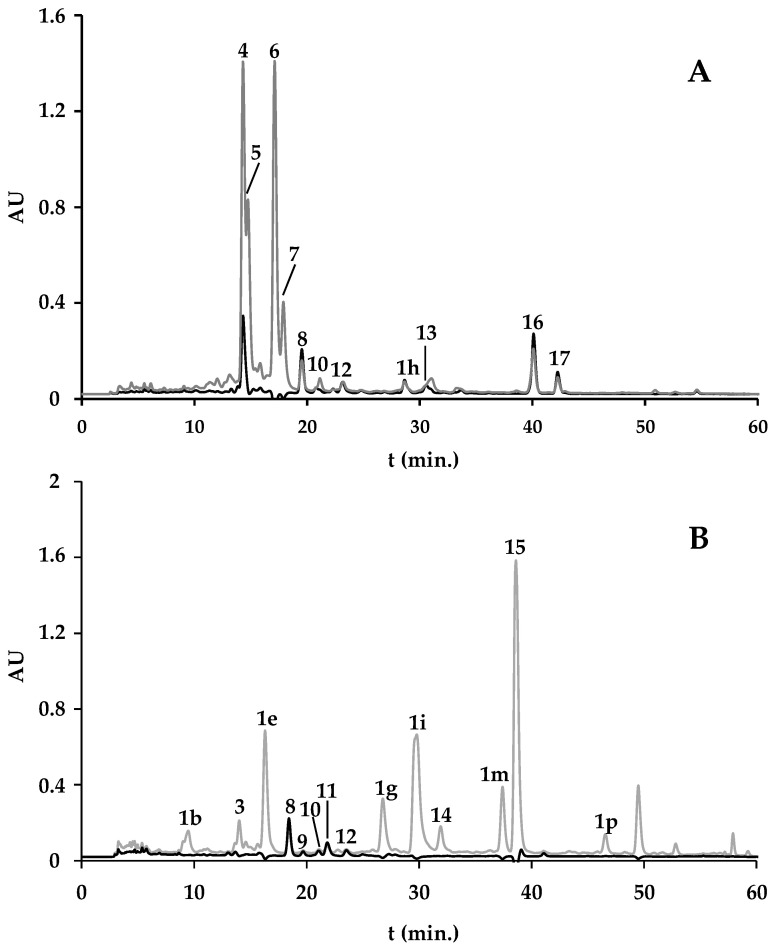
Representative HPLC-DAD chromatogram of the carotenoid and chlorophyll profiles of ethanol extracts obtained from *A. platensis* from A (**A**), spirulina-based supplement from C (**B**). Detection at 420 nm (gray line) and 450 nm (black line). Pheophytin *a* derivatives (**1b**, **1e**, **1g**–**1i**, **1m**, **1p**); chlorophyll *a* (**4**); zeaxanthin (**8**); unknown xanthophylls (**9**–**12**); unknown chlorophylls (**3**, **5**, **6**, **7**, **14**); unknown carotenes (**13**, **17**); β-carotene (**16**), and pheophytin *a* (**15**).

In an attempt to confirm the authenticity of the spirulina-based supplements, powdered *A. platensis*, obtained commercially from supplier A was used here as a control. Figure 1A shows the chromatographic analysis of the ethanol extract of spirulina purchased as powder revealed a pigment profile mainly composed by chlorophyll *a* (**4**), zeaxanthin (**8**), β-carotene (**16**), and pheophytin *a* (**15**) (Figure 1A). Other compounds were detected, but their complete identification was not achieved. Still, based on the analysis of their UV-Vis spectra and/or their elution order they were designated as unknown xanthophylls (**10**, **12**), unknown carotenes (**13** and **17**), unknown chlorophylls (**5**, **6**, and **7**), and an unknown pheophytin *a* derivative (**1h**) (Figure 1A). In a general way, except for the noted absence of chlorophyll *a* (**4**) in the ethanol extracts of spirulina-based supplements (from B and C) and the absence of β-carotene (**16**) in the ethanol extract of spirulina-based supplement from C, identical qualitative profiles were observed between ethanol extracts of spirulina-based supplements and powdered *A. platensis* (Figure 1, Table 1). Although most of the existing works report total content of carotenoids, determined spectrophotometrically, the presence of chlorophyll *a*, zeaxanthin, and β-carotene is in accordance with the pigment profile previously reported for spirulina [11,12]. Davani and colleagues [12] also reported the presence of compounds with UV-Vis profiles similar to chlorophylls and compounds with UV-Vis profiles similar to carotenoids, whose complete identity was not possible to be established, as verified for the samples analyzed herein (compounds **5**, **6**, **7**, and **1h**, and compounds **10** and **12**, respectively) (Figure 1, Table 1). As far as we know, pheophytin *a* (**15**) was now reported for the first time in spirulina. The presence of pheophytin *a*, a demetalated derivative of chlorophyll *a*, may indicate degradation of the samples, before, during, or after the production of the commercial spirulina powdered product. This suspicion is reinforced by the absence of chlorophyll *a* in the spirulina-based supplements and by the presence of several compounds without retention time correspondence but with a similar UV-Vis spectrum to the reference standard of a pheophytin *a*, being classified herein as pheophytin *a* derivatives (**1b**, **1e**, **1g**–**1i**, **1m**, **1p**) (Figure 1B, Table 1). We supposed that compounds **1b**, **1e**, **1g**–**1i**, **1m**, **1p** can be isomers, which show the same spectroscopic features, but different chromatographic performance. According to Anniva and colleagues [13], the presence and the content of pheophytin derivatives can represent an index in the quality control. These authors suggest their use as an index of oxidative deterioration and loss of freshness on storage, allowing insight into the history of food products [13]. Although lutein, cryptoxanthin, and chlorophyll *b* are also described in spirulina, these pigments were not found here.

A total content of 139.91, 106.48, and 87.45 mg/g dry ethanol extract was obtained for the powdered *A. platensis*, spirulina-based supplement from B, and spirulina-based supplement from C, respectively (Table 1). Since pheophytin *a* derivatives (**1b**, **1d**, **1e**, **1g**–**1i**, **1m**, **1p** and **1q**) were quantified as chlorophyll *a*, these compounds were presented as a sum (Table 1). Due to their UV-Vis spectra characteristic of chlorophylls and partially similar to that of chlorophyll *b*, with a maximum absorption at 652 nm, the unknown chlorophylls **2**, **3**, **5**, **6**, **7**, and **14**, were quantified as chlorophyll *b*, their sum being also presented in Table 1.

Chlorophyll *a* (**4**) was the major compound in the ethanol extract of *A. platensis* from A, representing ca. 28% of the total pigment content in this sample. The sum of all unknown chlorophylls represented more than 66% of the total pigments in the ethanol extract of *A. platensis* from A. The total content of these compounds was much lower in the ethanol extract of the spirulina-based supplement from C (5.95 mg/mg dry extract) (Table 1). On the other hand, this sample is the one with the highest content of pheophytin *a* and its derivatives (30.48 and 48.95 mg/g dry extract, respectively) (Table 1), reinforcing the idea of the occurrence of degradation phenomena during or after processing. In both supplements (from B and C), the content of pheophytin *a* derivatives was higher than that observed in the ethanol extract of *A. platensis* from A (Table 1).

Colla and collaborators [14] reported the easy degradation of *A. platensis*, measured by the decrease in its antioxidant potential, when exposed to heat, and this degradation could be greater than 50% after 30 days of exposure. These authors indicate that, due to the thermal and photolytic instability of some of its constituents, namely of phycocyanin, microalgae should be stored in packages that protect them from degradation processes, namely at temperatures below 10 °C in amber, hermetically sealed packages, especially when therapeutic indications are related to antioxidant activity [14]. Although the supplements studied here are sold in amber packaging, we are unaware of exposure to light and high temperatures during their production.

#### 2.1.2. *F. vesiculosus* and *F. vesiculosus*-Based Supplements

**Figure 2:**
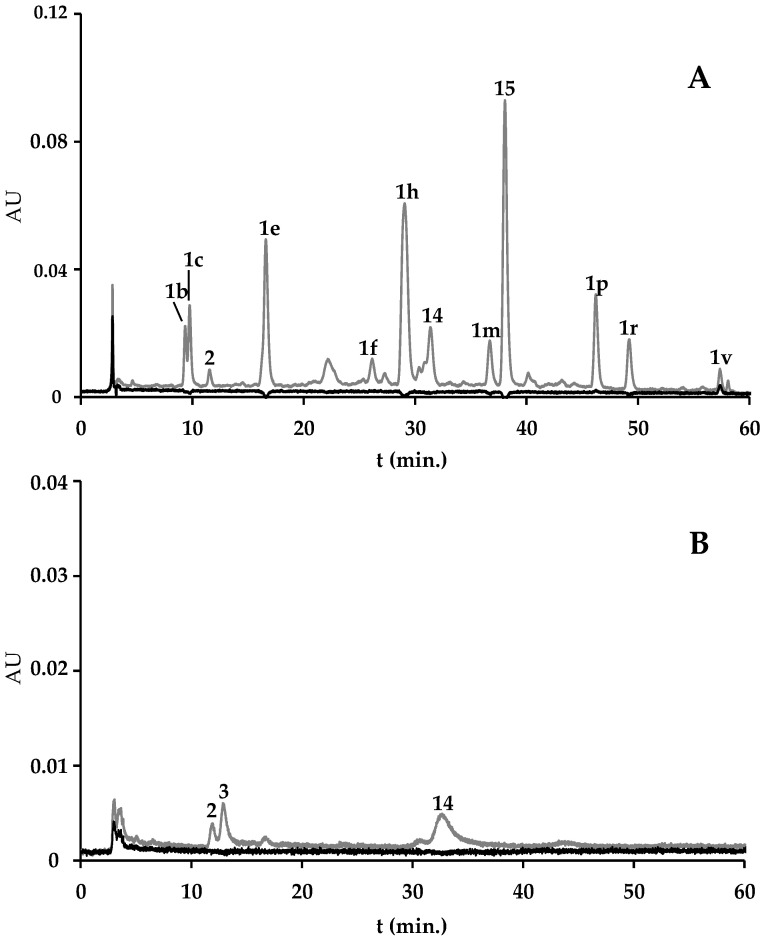
Representative HPLC-DAD chromatogram of the carotenoid and chlorophyll profiles of ethanol extracts obtained from wild *F. vesiculosus* (**A**) and from *F. vesiculosus*-based supplement from E (**B**). Detection at 420 nm (gray line) and 450 nm (black line). Pheophytin *a* derivatives (**1b**, **1c**, **1e**, **1f**, **1h**, **1m**, **1p**, **1r**, **1v**), unknown chlorophylls (**2**, **3**, **14**), and pheophytin *a* (**15**).

Seaweeds thrive in incredibly varied and ever-changing light conditions within their natural environments. This light climate serves as a crucial and intricate abiotic factor that significantly affects these organisms [15]. Additionally, factors beyond light, including sea surface temperature (SST), to which macroalgae are exposed throughout the seasons, can influence their metabolism, and subsequently change their chemical composition [9,16,17,18,19,20]. As the origin of the *F. vesiculosus* used in the production of the *F. vesiculosus*-based supplements studied herein is not mentioned in the product label, the profiles of *F. vesiculosus* pigments from different sources—from the sea (wild) (Figure 2A), from aquaculture, as well as a sample obtained commercially from an herbal shop (D)—were characterized. As the period established by law for the shelf life of a food supplement is between 3 to 5 years, and as the shelf life of *F. vesiculosus*-based supplements expires between 2022 and 2023, we chose to study a wild seaweed harvested in 2018 and deposited away from light and humidity in our laboratory ever since. The chromatographic analysis of the wild *F. vesiculosus* (Figure 2A), *F. vesiculosus* from aquaculture and *F. vesiculosus* from D, revealed a similar but surprising pigment profile, composed only by pheophytin *a* derivatives (**1a**–**1c**, **1d**, **1e**, **1f**–**1h**, **1k**, **1l**, **1m**, **1o**–**1q**, **1r**, **1s**, **1v**), unknown chlorophylls (**2**, **3**, **14**), and pheophytin *a* (**15**) itself (Table 1). Unexpectedly, fucoxanthin, the chemotaxonomic marker of Ochrophyta [10], was not found in any sample. According to previous works, this xanthophyll represents about 70% of the total xanthophyll content in *F. vesiculosus* [21,22]. Additionally, no other xanthophylls, such as violaxanthin, neoxanthin, and zeaxanthin, as well as chlorophylls *a* and *c* described for *F. vesiculosus* [21,22] were found in the studied samples. Some factors, such as pH, light exposure, oxidation-reduction reactions, and temperature, can contribute to fucoxanthin degradation [23]. It is known that heat treatment may also affect fucoxanthin content, its antioxidant activity, and even the color of the seaweed [23]. Fucoxantin instability is mainly due to its polyunsaturated structure. So, the observed chromatographic profile clearly shows degradation phenomena of *F. vesiculosus*, regardless of its origin. Although the specimens collected at sea were stored in conditions postulated to avoid the degradation (in a dry place and away from light), the degradation of the sample is notorious. This is even more evident in the supplements, which showed a poorer pigment profile: in the *F. vesiculosus*-based supplement from E, only unknown chlorophylls were found (compounds **2**, **3**, **14**) (Figure 2B) in trace amounts (Table 1), and only pheophytin *a* derivatives (compounds **1j**, **1n**, **1t**, **1u**, and **1v**) were found in the *F. vesiculosus*-based supplement from B (Table 1).

**Table 1:** Pigment content of the different algae-based supplements studied (from spirulina and/or *F. vesiculosus*), as well as of the commercial *Arthrospira platensis* (powdered *A. platensis*) and of *Fucus vesiculosus* (*F. vesiculosus*) from different sources (wild, aquaculture, and herbal shops) (mg/g dry extract) ^1^.

Algae Material/Supplement	Pheoph. *a* der. (1a–1v)	Chlor. *a* (4)	Unkn. Chlor. (2, 3, 5, 6, 7, 14)	Zeax. (8)	Unkn. Xant. (9–12)	β-Carot. (16)	Unkn. Carot. (13, 17)	Pheoph. A (15)	Total	Extraction Yield ^2^ (%)
*A. platensis* from A	3.04 (0.14)	39.13 (0.94)	92.86 (1.77)	1.42 (0.09)	nq	2.08 (0.35)	1.37 (0.24)	−	139.91 (0.14)	4.28 (0.48)
Spirulina-based supplement from B	9.99 (1.71)	−	94.87 (1.14)	0.82 (0.02)	nq	0.62 (0.12)	0.18 (0.03)	−	106.48 (2.99)	2.31 (0.12)
Spirulina-based supplement from C	48.95 (4.56)	−	5.95 (0.93)	0.95 (0.03)	1.11 (0.05)	−	−	30.48 (0.48)	87.45 (6.05)	5.79 (0.39)
*F. vesiculosus* wild	3.77 (0.08)	−	0.24 (0.01)	−	−	−	−	1.59 (0.08)	5.82 (0.30)	4.20 (0.13)
*F. vesiculosus* from aquaculture	22.10 (1.46)	−	−	−	−	−	−	−	22.10 (1.46)	3.76 (0.14)
*F. vesiculosus* from D	9.90 (0.28)	−	−	−	−	−	−	−	9.90 (0.28)	2.78 (0.04)
*F. vesiculosus*-based supplement from B	4.34 (0.66)	−	−	−	−	−	−	−	4.34 (0.66)	3.28 (0.04)
*F. vesiculosus*-based supplement from E	nq	nq	nq	−	−	−	−	−	nq	*
*F. vesiculosus* & Spirulina-based supplement from F	21.30 (2.87)	−	86.21 (3.25)	0.97 (0.15)	nq	0.19 (0.00)	3.52 (1.79)	−	108.75 (4.94)	4.48 (0.28)

^1^ Results are expressed as mean (standard deviation) of three determinations; nq: not quantified; “−”: not detected; Chlor.: chlorophyll; Unkn: unknown; Xant.: xantophylls; Carot.: carotene(s); Pheoph.: pheophytin; Zeax.: zeaxanthin; ^2^ Calculated as mass of dried extract/mass of dried sample × 100 (%); * liquid sample, not subjected to extraction process.

In quantitative terms, the *F. vesiculosus* from aquaculture was the richest material, presenting a total pigment content of 22.10 mg/g dry extract (Table 1). Higher levels of pigments in algae from aquaculture compared to algae of marine origin had already been reported by us previously [9].

#### 2.1.3. *F. vesiculosus* and Spirulina-Based Supplement

The supplement containing a mix of *F. vesiculosus* and spirulina (from F) revealed a pigment profile with a similarity of ca. 78% to that of the powdered spirulina ethanol extract (Table 1). Of the nine compounds identified in this supplement (**3**, **5**, **1d**, **8**, **10**, **1h**, **13**, **16**, and **17**), only two (**1d** and **1h**) are common to both the *F. vesiculosus* and spirulina-based supplement and to *F. vesiculosus* (from wild or aquaculture origin). This pigment profile was expected, since, according to the product label, this supplement contains *F. vesiculosus* + spirulina in a ratio of 1:28 mg (*F. vesiculosus*: spirulina) per capsule.

The total pigment content of the *F. vesiculosus* and spirulina-based supplement was also very similar to that found for *A. platensis* ethanol extract (108.75 mg/g dry basis) (Table 1). The unknown chlorophylls were the predominant compounds, representing ca. 79% of total pigments quantified in this extract (Table 1).

#### 2.1.4. Labeling Analysis

Table 2 presents the pharmaceutical formulation, the instructions for consumption and the recommended daily dose, according to the analysis of the label of each product. The daily consumption of pigments associated with the recommended daily dose, calculated according to the extraction yield obtained for each analyzed sample, was also included in Table 2.

Although the consumption of food supplements of natural origin is an increasingly attractive option in the search for solutions to overcome nutritional deficiencies or as a modern, effective, and healthy way to lose weight, food supplements for weight loss can result in benefits or dangers, depending on the good or bad management of its consumption. In fact, with natural products it is often not clear what the optimal doses are to balance efficacy and safety. Preparation of products may vary from manufacturer to manufacturer, and from batch to batch within one manufacturer. Because it is often not clear what the active component(s) of a product is, standardization may not be possible, and the clinical effects of different brands may not be comparable [24].

Despite the beneficial health effects associated with all of the metabolites identified here, the possible negative effects of regular consumption of high doses and their consequent bioaccumulation in the human body, as well as the possibility of drug interactions, cannot be ignored. For instance, according to the available research, it appears that chlorophyll is generally safe and without many side effects or toxicities in nonsensitive people [25,26,27]. Nevertheless, adverse effects, usually gastrointestinal or of dermatologic nature, have already been reported [24]. Its excessive consumption by people with hypersensitivity to chlorophyll or any of its metabolites can lead to more serious effects, including cardiovascular, hematologic, genitourinary, and renal effects, among others [24].

**Table 2:** Pharmaceutical formulation, instructions for consumption and the estimated daily content of pigments ingested according to the recommended daily dose included in the label of each commercial product (mg/g sample) ^1^.

Algae Material/Supplement	Pharmaceutical Formulation	Instructions for Consumption/ Recommended Daily Dose	Daily Content (mg) of the Compounds Ingested Following the Supplier’s Recommendations					
			Chlor. *a*	Zeax.	β-Carot.	Pheoph. *a*	Pheoph. *a* der.	Unkn. Chlor.
*A. platensis* from A	Powder	1 teaspoon/day	2.24 (0.05)	0.08 (0.01)	0.12 (0.02)	−	0.17 (0.01)	5.32 (0.10)
Spirulina-based supplement from B	Capsules	2 capsules, 3 times/day	−	0.07 (0.00)	0.05 (0.01)	−	0.75 (0.12)	7.95 (0.93)
Spirulina-based supplement from C	Pills	4 pills/day	−	0.23 (0.01)	−	7.37 (0.12)	11.84 (1.10)	1.44 (0.23)
*F. vesiculosus*-based supplement from B	Pills	2 pills, 3 times/day	−	−	−	−	0.51 (0.09)	−
*F. vesiculosus*-based supplement from E	Drops	30 drops, 3 times/day	nq	−	−	−	−	nq
*F. vesiculosus* & Spirulina-based supplement from F	Capsules	2 capsules, 3 times/day	−	0.13 (0.02)	0.03 (0.00)	−	2.77 (0.37)	11.22 (0.42)

^1^ Results are expressed as mean (standard deviation) of three determinations; “−”: not found; Chlor.: chlorophyll; Zeax.: zeaxanthin; Unkn: unknown; Carot.: carotene; Pheoph.: pheophytin.

### 2.2. Total Phenolic Content (TPC)

The TPC found in ethanol extracts of the studied samples ranged from 6.50 to 12.33 µg GAE/mg extract (Table 3). In general, the *F. vesiculosus* and *F. vesiculosus*-based supplements presented higher TPC, with the *F. vesiculosus* from aquaculture and *F. vesiculosus*-based supplement from E being the richest material, with 11.34 and 12.33 µg GAE/mg extract, respectively. Contrarily, the ethanol extract obtained from powdered *A. platensis* (from A) and spirulina-based supplements presented similar TPC (Table 3). 

The higher content of TPC found in *F. vesiculosus* samples is certainly related with the presence of phlorotannins, marine polyphenols exclusively produced by brown seaweeds [28].

**Table 3:** TPC of the different algae-based supplements studied (from spirulina and/or *F. vesiculosus*), as well as of the commercial powdered *A. platensis* (*A. platensis* from A) and of *F. vesiculosus* from different sources (wild, aquaculture, and herbal shops) ^1,2^.

Sample	TPC (µg GAE/mg Dry Extract)
*A. platensis* from A	6.50 ± 0.45
Spirulina-based supplement from B	6.85 ± 0.41
Spirulina-based supplement from C	7.80 ± 0.78
*F. vesiculosus* wild	8.96 ± 0.92
*F. vesiculosus* aquaculture	11.34 ± 0.02
*F. vesiculosus* from D	7.0 ± 0.39
*F. vesiculosus*-based supplement from B	6.56 ± 0.43
*F. vesiculosus*-based supplement from E	12.33 ± 0.19
*F. vesiculosus* & spirulina-based supplement from F	7.68 ± 0.29

^1^ GAE—Gallic acid equivalents. ^2^ Values are expressed as the mean ± SD of three independent experiments performed in duplicate.

### 2.3. Biological Assays

#### 2.3.1. Metabolism of Carbohydrates

Carbohydrates are typically the primary source of calories in most diets. Before being absorbed by the body, carbohydrates are broken down into monosaccharides. This occurs due to two major enzymes: α-amylase and α-glucosidase. α-Amylase catalyzes the hydrolysis of α-(1,4)-d-glycosidic linkages of starch and other glucose polymers [29]. On the other hand, α-glucosidase is present in the small intestine, promoting a delay in the glucose absorption [30]. Inhibition of this digestion or absorption can effectively reduce caloric intake, thereby facilitating weight loss and helping to fight obesity.

**Figure 3:**
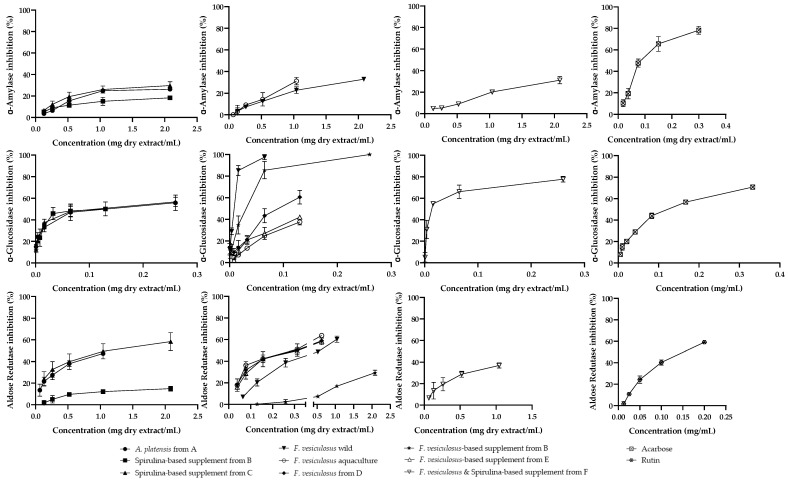
α-Amylase, α-glucosidase, and aldose reductase inhibition by the analyzed samples and by positive controls (acarbose and rutin). Results are expressed as mean ± SD of three independent experiments, each performed in triplicate.

Although to different extents and behaviors, all studied extracts showed a capacity to inhibit the activity of both *α*-amylase and *α*-glucosidase in a concentration-dependent manner (Figure 3). A more effective action was observed towards *α*-glucosidase. The maximum *α*-amylase inhibitory activity did not go beyond 33%, at the highest extract concentrations tested (Figure 3). IC_25_ values were determined in order to compare the activity exhibited for all extracts, as it was not possible to reach 50% activity for all samples. 

Ethanol extracts of spirulina-based supplements exhibited an α-glucosidase inhibitory effect similar to that observed for the ethanol extract of powdered *A. platensis* (from A) (IC_25 Spirul. A_ = 6.96 ± 0.84 µg/mL; IC_25 Spirul. supp. B_ = 10.17 ± 0.95 µg/mL; IC_25 Spirul. supp. C_ = 7.77 ± 1.12 µg/mL) (Figure 3). All extracts showed at least a 3-fold higher inhibitory capacity towards α-glucosidase than acarbose (IC_25_ = 30.53 ± 2.42 µg/mL), the positive control used for this assay.

Concerning α-amylase, a similar inhibitory effect was observed for ethanol extracts obtained from *A. platensis* and from the spirulina-based supplement from C (maximum activity of ca. 26% and 30%, respectively), for the highest concentration tested (2 mg dry extract/mL) (Figure 3). For this enzyme, the spirulina-based supplement from B was less active (ca. 18% of inhibition at 2 mg dry extract/mL). The capacity to inhibit α-amylase observed for all extracts was far below that of the commercial inhibitor acarbose (IC_25_ = 41.33 ± 0.58 µg/mL), the positive control used in this assay.

Concerning the *F. vesiculosus*-related samples, with the exception of the ethanol extract of *F. vesiculosus*-based supplement from B (IC_25 F.ves. supp. B_ = 10.99 ± 4.89 µg/mL), all *F. vesiculosus* products showed a lower α-glucosidase inhibitory effect than *F. vesiculosus* from the sea (IC_25 F.ves. wild_ = 30.59 ± 0.08 µg/mL; IC_25 F.ves. D_ = 37.73 ± 8.78 µg/mL; IC_25 F.ves. aquac._ = 66.29 ± 2.86 µg/mL; IC_25 F.ves. supp. E_ = 66.73 ± 5.91 µg/mL) (Figure 3). The *F. vesiculosus*-based supplement from B was more effective than the positive control while *F. vesiculosus* from the sea and the remain *F. vesiculosus*-related samples showed IC values of only up to two times higher than that obtained with acarbose (IC_25 acarbose_ = 30.53 ± 2.42 µg/mL) (Figure 3). On the other hand, only the ethanol extracts obtained from *F. vesiculosus* from the sea, from aquaculture, as well as from the *F. vesiculosus*-based supplement from E exhibited some activity towards α-amylase, the last one the less potent (ca. 33%, 31%, and 24% of activity, respectively, at 2 mg dry extract/mL). These results show that not all supplements have the same effectiveness.

The ethanol extract obtained from the *F. vesiculosus* and spirulina-based supplement from F showed higher α-glucosidase inhibitory activity (IC_25_ = 4.54 ± 0.81 µg/mL) than that exhibited by powdered *A. platensis* and *A. platensis*-based supplements, as well as by *F. vesiculosus* and any one of the *F. vesiculosus*-based supplements herein studied (Figure 3). This result can probably be due to the synergic events between the chemical composition of both spirulina and *F.vesiculosus* constituents. Concerning α-amylase, 2 mg of the ethanol extract of the *F. vesiculosus* and spirulina-based supplement/mL led to a maximum inhibition of ca. 31% of this enzyme, similar to what was observed for the extracts of *A. platensis* and *F. vesiculosus*-related materials that exhibited activity (Figure 3). The inhibitory activities of *A. platensis* and *F. vesiculosus* for α-glucosidase and α-amylase, widely known and more pronounced to that observed here, are mainly attributed to their chemical composition, namely phycocyanin [31] and phlorotannins [32,33,34]. In fact, it is important to emphasize that in extracts from such complex natural matrices it is hard to identify all of the compounds. Synergic and/or antagonic effects between all the constituents of an extract must be considered. However, as observed with pigment composition, the degradation of other existing metabolites should be considered. The results obtained here show that algae-based supplements may interfere with the absorption of carbohydrates; however, the moderate activity observed suggests caution in ensuring their properties for weight loss.

#### 2.3.2. Aldose Reductase

Aldose reductase is a key enzyme of the polyol pathway that can reduce glucose to sorbitol in the presence of NADPH. As reported in a recent work [35], aldose reductase mediates lipid accumulation in the murine heart and liver. These data unveil new opportunities to target this pathway to fight obesity [35]. 

All ethanol extracts studied herein were able to inhibit the aldose reductase activity in a concentration-dependent manner (Figure 3). However, the maximum inhibitory activity observed does not exceed 60%. The most pronounced inhibitory effect was observed for the ethanol extracts of *F. vesiculosus*-related samples. Ethanol extracts of the *F. vesiculosus* from aquaculture, as well as *F. vesiculosus*-based supplements from D, and from E showed IC_25_ values close to that obtained for the positive control (IC_25 F.ves. aquac._ = 53.74 ± 8.42 µg/mL; IC_25 F.ves. D_ = 61.00 ± 6.83 µg/mL, IC_25 F.ves. supp. E_ = 72.39 ± 9.88 µg/mL, IC_25 rutin_ = 52.10 ± 7.17 µg/mL) (Figure 3). The ethanol extract obtained from the *F. vesiculosus*-based supplement from B was the least active sample (IC_25_ = 1737.96 ± 98.26 µg/mL) (Figure 3), distinguishing itself from other *F. vesiculosus*-based supplements.

The supplement containing spirulina from B was also the least active among the spirulina products evaluated (IC_25 Spirul. A_ = 155.95 ± 52.03 µg/mL; IC_25 Spirul. supp. C_ = 203.29 ± 92.16 µg/mL). This extract was only able to inhibit aldose reductase up to 14.9 ± 2.2%, at the maximum tested concentration (2.0 mg/mL).

There was no increase in activity resulting from the mixture of *F. vesiculosus* and spirulina (IC_25_ = 335.12 ± 62.54 µg/mL) (Figure 3). 

Similar to what was observed for α-glucosidase and α-amylase enzymes, a reduction of the capacity to inhibit aldose reductase was also observed herein, considering what was already described for *A. platensis* and *F. vesiculosus* species [31,32,33,34].

#### 2.3.3. Antiradical Activity

Many obesity-related diseases are often associated with oxidative stress, which could potentially serve as the underlying mechanism for the initiation or progression of these conditions. Several studies have established a correlation between biomarkers of oxidative stress and obesity [3,4]. Obese individuals show higher concentrations of plasma isoprostanes, plasma malondialdehyde, skeletal muscle 4-hydroxynonenal, and plasma protein carbonyls [4]. The capacity of *F. vesiculosus*, *A. platensis*, and their related products to scavenge O_2_^•−^ and ^•^NO was herein evaluated.

**Figure 4:**
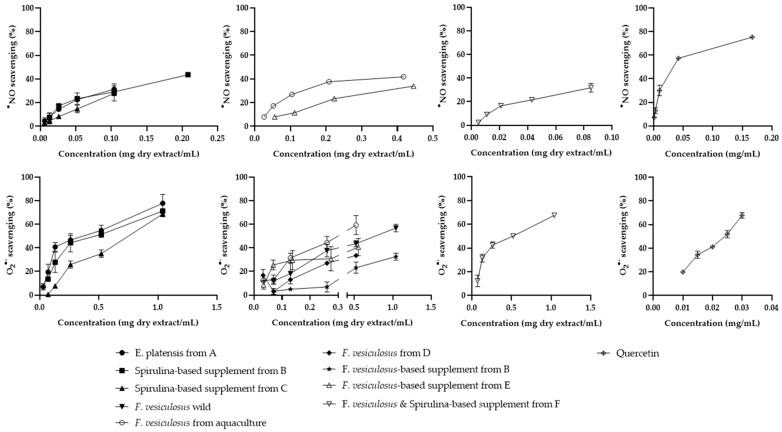
^•^NO and O_2_^•−^ scavenging activity by the analyzed samples and by the positive control (quercetin). Results are expressed as mean ± SD of three independent experiments, each performed in triplicate.

The ethanol extracts obtained from algae and algae-based products were able to intercept the in vitro-generated reactive species O_2_^•−^ and ^•^NO in a concentration-dependent way (Figure 4). Better results were observed for O_2_^•−^ scavenging (Figure 4). Overall, the extracts showed distinct scavenging behavior for the two assays (Figure 4).

The ethanol extract of the spirulina-based supplement from C was the most promising for the O_2_^•−^ (IC_25_ = 67.16 ± 10.14 µg/mL), but only showed a maximum ^•^NO scavenging capacity of ca. 24% for the highest concentration tested (104 µg dry extract/mL). The ethanol extract of the spirulina-based supplement from B was less potent for O_2_^•−^ (IC_25_ = 122.84 ± 34.14 µg/mL) but more effective against ^•^NO (IC_25_ = 49.19 ± 10.20 µg/mL) (Figure 4). 

Concerning the *F. vesiculosus*-related samples, the ethanol extracts of the *F. vesiculosus*-based supplement from E and *F. vesiculosus* from aquaculture were the most potent against O_2_^•−^ (IC_25_ = 80.28 ± 5.36 and 91.98 ± 15.35 µg/mL, respectively) (Figure 4). These were the only *F. vesiculosus*-related products that showed activity against ^•^NO, although in this assay, better results were observed for the ethanolic extract of *F. vesiculosus* from aquaculture (IC_25 F.ves. aquac._ = 87.94 ± 2.12 µg/mL; IC_25 F.ves. supp. E_ = 211.80 ± 37.08 µg/mL) (Figure 4).

There was no increase in the activity resulting from the mixture of *F. vesiculosus* and spirulina. This extract showed an IC_25_ value of 116.26 ± 25.63 µg/mL for O_2_^•−^ and just a maximum activity of ca. 32% against ^•^NO at 85 µg dry extract/mL (the highest concentration tested) (Figure 4). None of the extracts studied showed activity close to that observed for quercetin, the positive control used for both O_2_^•−^ and ^•^NO assays (IC_25_ = 11.71 ± 0.58 µg/mL and 7.82 ± 1.66 µg/mL, respectively) (Figure 4).

The potent antiradical capacity reported for *F. vesiculosus* and spirulina [36,37] was not observed herein for the analyzed samples; however, the decrease of the antiradical potential can be somewhat related with the degradation of the original compounds of the samples.

## 3. Materials and Methods

### 3.1. Standards and Reagents

The standard of pheophytin *a* (90.0%) was from LGC Standards (Irvine, CA, USA). Standards of fucoxanthin (≥95.0%), β-carotene (≥95.0%), chlorophyll *a*, and chlorophyll *b*, sodium phosphate, sodium nitroprusside dihydrate (SNP), β-nicotinamide adenine dinucleotide reduced form (NADH), phosphoric acid H_3_PO_4_), sulphanilamide, phenazine methosulphate (PMS), monopotassium phosphate (KH_2_PO_4_), nitroblue tetrazolium (NBT), ethanol, p-nitrophenyl-α-glucopyranoside (NGP), α-glucosidase from Saccharomyces cerevisiae, monopotassium phosphate (KH_2_PO_4_), trisodium phosphate (Na_3_PO_4_), hydrochloride acid (HCl), starch, α-amylase from porcine pancreas, dinitrosalicylic acid (DNS), sodium hydroxide (NaOH), potassium sodium tartrate tetrahydrate (KNaC_4_H_4_O_6_.4H_2_O), D,L-glyceraldehyde, β-nicotinamide adenine dinucleotide phosphate reduced form (NADPH), 2,6-di-*tert*-butyl-4-methylphenol (BHT), and methyl *tert*-butyl ether (MTBE), were obtained from Sigma-Aldrich (St. Louis, MO, USA). Standards of zeaxanthin (97.0%), lutein, and β-cryptoxanthin were obtained from CaroteNature (Lupsinggen, Switzerland). Human aldose reductase was acquired from Prozomix (Northumberland, UK). N-(1-naphthyl) ethylenediamine dihydrochloride and methanol Lichrosolv were from Fisher Chemical (Loughborough, UK). Tetrahydrofuran (THF) was acquired from Merck (Darmstadt, Germany).

### 3.2. Sampling

The samples analyzed in this study were obtained from different sources (Table 4). Except for the wild *F. vesiculosus*, all samples were commercially obtained. Powdered (≤910 μm) *A. platensis* from the supplier A, spirulina-based supplements from two distinct herbal suppliers (B and C), dried *F. vesiculosus* from supplier D, *F. vesiculosus*-based supplements from two distinct herbal suppliers (B and E) and one supplement containing a mix of *F. vesiculosus* and spirulina (*F. vesiculosus* & Spirulina) from supplier F, were purchased in an herbal shop, in November 2021. *F. vesiculosus* from aquaculture was purchased from an integrated cultivation company with quality guarantees in December 2021. Wild *F. vesiculosus* was collected at Praia Norte beach (N 41°41′49.3″, W 8°51′3.39″), Viana do Castelo (Portugal), in December 2018, and identified by Graciliana Lopes, (Ciimar, Portugal). The collected specimens were immediately transported to the laboratory in insulated ice boxes. The sample, corresponding to a pool of five individuals in the same stage of development, was washed with NaCl aqueous solution (3.5%, *w*/*v*) to remove epiphytes and encrusting material, freeze-dried (Virtis SP Scientific Sentry 2.0 apparatus, Gardiner, NY, USA) and then lyophilized. *F. vesiculosus* samples (from the sea, from aquaculture and from supplier D) were powdered (≤910 μm) and stored in a desiccator, in the dark, until extraction. Voucher specimens, labeled in Table 4, were deposited at Laboratório de Farmacognosia, Faculdade de Farmácia, Universidade do Porto (Portugal).

### 3.3. Extraction

Extracts were prepared with ca. 2 g of each dried algae/algae-based supplement, using 100 mL of ethanol, under the following conditions: 5 min of sonication followed by 30 min of stirring maceration (800 rpm), at room temperature. Each sample was extracted three times. The obtained extracts were gathered, filtered under vacuum, and evaporated at reduced pressure (Rotavapor R-215, BÜCHI Labortechnik, Flawil, Switzerland) until complete dryness. The dried extracts were kept at −20 °C and protected from light until analysis.

### 3.4. Chemical Analysis

#### 3.4.1. HPLC-DAD Analysis

The dried residue of each extract was redissolved in 100% ethanol, sonicated, filtered through 0.45 μm size pore membrane (Millipore, Bedford, MA, USA) and then analyzed on an analytical HPLC unit (Gilson Medical Electronics, Villiers le Bel, France), equipped with a C30 YMC carotenoid column 5 μm, 250 × 4.6 mm (YMC Co., Ltd., Kyoto, Japan) maintained at 26 °C, following the procedure described by Les [38]. Briefly, the mobile phase constituted by two solvents, methanol (A) and *tert*-butyl methyl ether (B), started with 95% A and used a gradient to obtain 70% at 30 min, 50% at 50 min, 0% at 60 min, 0% isocratic at 65 min, and 95% at 70 min. The flow rate was 0.9 mL/min. Detection was achieved with an Agilent 1100 series diode array detector (DAD) (Agilent Technolo-gies, Waldbronn, Germany). Spectral data from all peaks were collected in the range of 200–700 nm, and chromatograms were recorded at 450 nm. The data were processed on Unipoint System software (http://www.hplc.ru/catalogs/Gilson_unipoint_info.pdf, accessed on 1 December 2023; Gilson Medical Electronics, Villiers le Bel, France). The compounds were identified by comparing their retention times and UV-Vis spectra in the range of 200–700 nm with those of authentic standards.

For quantification purposes, 20 μL of each extract from algae/algae-based supplements were injected in triplicate. Peak purity was checked by the software contrast facilities and carotenoids and chlorophyll quantification was achieved by the absorbance recorded in the chromatograms at 450 and 420 nm, respectively, relative to external calibration curves. Chlorophyll *a* (**4**), zeaxanthin (**6**) and β-carotene (**15**) were quantified as themselves. Unknown xanthophylls (**9**–**12**) and unknown carotenes (**13**, **16**) were quantified as zeaxanthin and β-carotene, respectively. Pheophytin *a* (**17**) and pheophytin *a* derivatives (**1a**–**1v**) were determined as chlorophyll *a* and unknown chlorophylls (**2**, **3**, **5**, **7**, **8**, **14**) were quantified as chlorophyll *b*.

#### 3.4.2. Linearity

The linearity range of the method was assessed by building calibration curves, using five different concentration levels of the pure standards, according to the range of concentrations present in the samples (Table 5).

#### 3.4.3. Total Phenolic Content

TPC of the extracts was determined using the Folin–Ciocalteu assay, as in Morone and colleagues [39]. A standard calibration curve (*y* = 1.0384*x* + 0.2112; *r*^2^ = 0.9489) was obtained with seven concentrations of gallic acid (GA) (0.002 to 0.034 mg/mL). Total phenols in each extract were expressed in mg gallic acid equivalents (GAE)/g dry biomass. Three experiments were carried out in duplicate.

### 3.5. Biological Assays

#### 3.5.1. α-Glucosidase Inhibition

The effect of the extracts at inhibiting α-glucosidase activity was determined according to Barbosa and colleagues [40]. Three independent experiments were performed in triplicate. Acarbose was selected as the positive control.

#### 3.5.2. α-Amylase Inhibition

The extract capacity to modulate α-amylase activity was evaluated according to [40]. Three independent experiments were performed in triplicate. The pharmacological inhibitor acarbose was selected as the positive control.

#### 3.5.3. Aldose Reductase Inhibition

The potential of the extracts to inhibit aldose reductase was evaluated as in Barbosa and colleagues [40]. Three independent experiments were performed in triplicate. Rutin was used as positive control.

#### 3.5.4. Superoxide Anion Radical Scavenging

The superoxide scavenging (O_2_^•−^) activity of the extracts was assessed according to a previously reported protocol [40]. Three independent experiments were performed in triplicate. Quercetin was used as the positive control.

#### 3.5.5. Nitric Oxide Radical Scavenging

The nitric oxide scavenging (^•^NO) activity of the extracts was determined by the Griess reaction as in Pereira and co-workers [40]. Three independent experiments were performed, each one performed in triplicate. Quercetin was used as the positive control.

#### 3.5.6. Data Processing

Data analysis, including IC_25_ values calculation, was carried out using GraphPad Prism 8 Software, Inc. (San Diego, CA, USA) for Windows.

## 4. Conclusions

This work attempts to provide information about the authenticity and effectiveness of several algae-based supplements indicated for weight loss for which there were no studies until now. Despite the apparent degradation of the samples, highlighted by the absence of fucoxanthin, a chemotaxonomic marker of brown algae that was not found in *F. vesiculosus* materials, it was possible to establish a coincident chemical profile between raw material and related commercial products. Our results show the effect of the algae-based supplements on the reduction of carbohydrate digestion or absorption through their capacity to inhibit α-glucosidase and α-amylase activities. Additionally, a moderate inhibitory effect towards aldose reductase and antiradical activity points out their potential as therapeutic adjuncts to address obesity. However, the general moderate activity observed suggests caution in assuring their properties for weight loss. 

Further work using in vivo models will ascertain the efficacy and safety of consumption of these supplements. Nevertheless, given the lack of existing legislation, the present work opens the door to the relevance of monitoring the general use of dietary supplements (sometimes replacing a healthy diet, or taken together with conventional drugs that may affect the dosage and the effect with associated toxicity).

## Figures and Tables

**Table 4:** Details of the studied samples.

Sample	Origin	Data of Collection */Purchase	Voucher Label
*A. platensis* powdered	Supplier A	November 2021	Artplat_A_Nov21
Spirulina-based supplement	Supplier B		Spir-Supplem_B_Nov21
Spirulina-based supplement	Supplier C		Spir-Supplem_C_Nov21
Wild *F. vesiculosus*	Praia Norte	December 2018	Fves_Wild_Dec21
*F. vesiculosus* from aquaculture	AlgaPlus	December 2021	Fves_Aquac_Dec21
Commercial *F. vesiculosus*	Supplier D	November 2021	Fves_D_Nov21
*F. vesiculosus*-based supplement	Supplier B		Fvesic-Supplem_B_Nov21
*F. vesiculosus*-based supplement	Supplier E		Fves-Supplem_E_Nov21
*F. vesiculosus* & Spirulina-based supplement	Supplier F		Fves&Spir-Supplem_F_Nov21

* Applied only for *F. vesiculosus* collected at sea (wild *F. vesiculosus*).

**Table 5:** Regression equation, r^2^ values and linearity range of the reference compounds used for pigments quantification.

Compound	Regression Equation (mg/mL)	*r* ^2^	Linearity (mg/mL)
Chlorophyll *a*	*y* = 9.72 × 10^8^*x* + 1149069.1	0.995	0.00078–0.100
Chlorophyll *b*	*y* = 8.94 × 10^8^*x* + 2137228.1	0.997	0.00078–0.100
Zeaxanthin	*y* = 3.91 × 10^9^*x* + 1172649.0	0.999	0.00041–0.106
β-Carotene	*y* = 4.33 × 10^9^*x* + 2869689.8	0.999	0.0001–0.100

## Data Availability

The data presented in this study are available for a limited time upon request from the corresponding author.
